# Transforaminal percutaneous endoscopic discectomy for symptomatic gas-filled discal cysts—report of three cases and literature review

**DOI:** 10.1186/s13018-021-02403-8

**Published:** 2021-04-13

**Authors:** Kejun Zhu, Dengwei He

**Affiliations:** Department of Spinal Surgery, Lishui Municipal Central Hospital, 289 Kuocang Road, Lishui, Zhejiang, 323000 People’s Republic of China

**Keywords:** Discal cyst, Gas-filled, Transforaminal percutaneous endoscopic discectomy, Intraspinal

## Abstract

**Objective:**

The aim of this retrospective study is to review our experience in the diagnosis and role of transforaminal percutaneous endoscopic discectomy (TPED) for symptomatic gas-filled discal cysts.

**Methods:**

Between May 2014 and June 2017, 3 patients from Lishui Center Hospital (Lishui China), who underwent TPED for symptomatic gas-filled discal cysts, were analyzed. The clinical features, imaging findings, operative findings, and treatment outcomes are presented. In addition, relevant literature regarding gas-filled discal cysts was searched using PubMed, and their characteristics, clinical features, therapeutic strategies, and survival outcomes were reviewed.

**Results:**

The median age of the patients was 56.7 years (range, 55–60 years). In all patients, a discal cyst was located in the lumbar region, and the patients presented with backache and numbness in the lower extremities. The diagnosis was made by lumbar 3-dimensional computed tomography (3D-CT) or magnetic resonance imaging (MRI). All patients underwent TPED. All patients recovered successfully and were eventually discharged. Eighteen articles were identified from the searches of the database, and a total of 42 patients were included. There were 28 males and 14 females. The mean age was 56.8 years, ranging from 27 to 85 years. Lower back pain was the major symptom. Twenty-two patients underwent surgery, 4 patients underwent percutaneous needle aspiration, 2 patients underwent drug therapy, 13 patients received nonoperative treatment, and 1 patient was unknown.

**Conclusion:**

TPED for gas-filled discal cysts is feasible, effective, and successful, although it should be performed by an experienced surgeon with awareness of the potential risk of severe nerve root injury.

## Introduction

Lumbar radiculopathy can be the result of many different and complicated pathogeneses that cause severe compression of the nerve root [1]. A rare cause of lumbar radiculopathy is discal cyst [1]. Discal cysts are intraspinal extradural cysts with a direct connection with the corresponding intervertebral disc [2]. To the best of our acknowledge, discal cysts of the lumbar spine are rare lesions. Furthermore, discal cysts that contain gas are extremely rare. Forty-three articles have been published describing approximately 105 cases of discal cysts in the available literature [3]. There are no detailed case reports concerning clinical course, imaging findings, diagnosis, and management strategies of gas-filled discal cysts. The main aims of the present study were to assess transforaminal percutaneous endoscopic discectomy (TPED) of symptomatic gas-filled discal cysts and to systematically review the previously reported cases in the literature.

## Patients and methods

We retrospectively reviewed the records of 3 patients with symptomatic gas-filled discal cysts who had undergone TPED at the Department of Spinal Surgery, Lishui Center Hospital, Lishui, China, between January 2014 and January 2018. Age, sex, medical history, location of lesions, clinical presentation, diagnostic methods, intraoperative findings, postoperative complications, and outcomes were retrieved from hospital records. Relevant literature and studies regarding gas-filled discal cysts were searched in “PubMed” and “Web of Science” from January 1990 to January 2019. The text words and MESH terms “gas,” “cyst,” “disc,” and “intraspinal” were used. Disease characteristics, clinicopathologic features, therapeutic strategies, and survival outcomes were reviewed, and the data were tabulated.

### Surgical strategy

The patient underwent TPED under local anesthesia, in the right lateral decubitus position. To increase the space of the interlaminar window, the hip and knee were flexed at 90° and 45°, respectively. A 7-mm incision was made on the skin. A catheter was inserted into the left intervertebral foramen at level 4/5, and a C-arm X-ray machine was positioned to obtain the proper views. After placing the dilated catheter, the working channel was established and connected with the light source of the endoscope. Normal saline flowed continuously to rinse the area. Following hemostasis, a bipolar radiofrequency knife head under the working channel was used to remove the discal cyst with the nucleus pulposus. A lumbar 5 nerve root canal expansion was performed.

## Results

### Report of cases

**Fig. 1:**
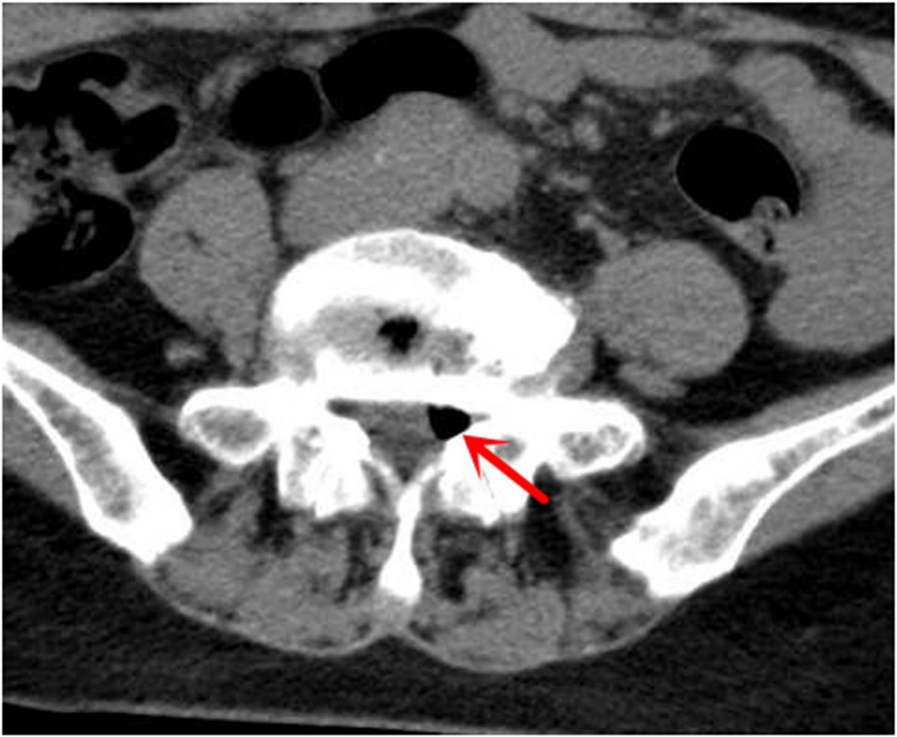
Computed tomography scan reveals low-density mass (red arrow)

**Fig. 2:**
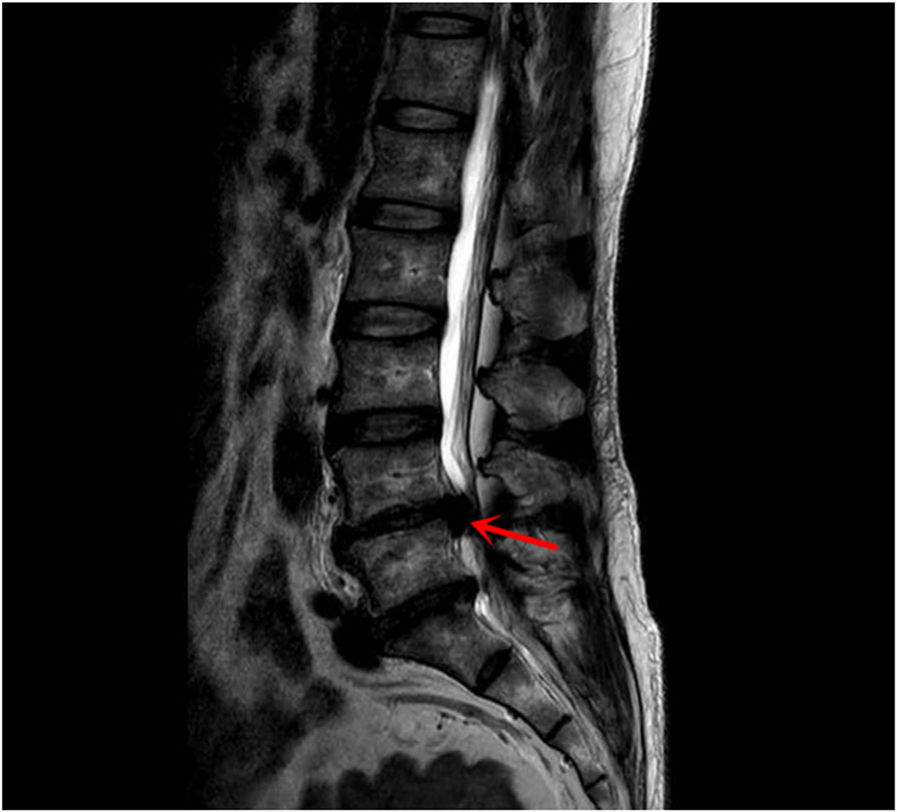
MRI imaging of the lumbar spine demonstrating a discal cyst with hypointense on T2-weighted images (red arrow)

**Fig. 3:**
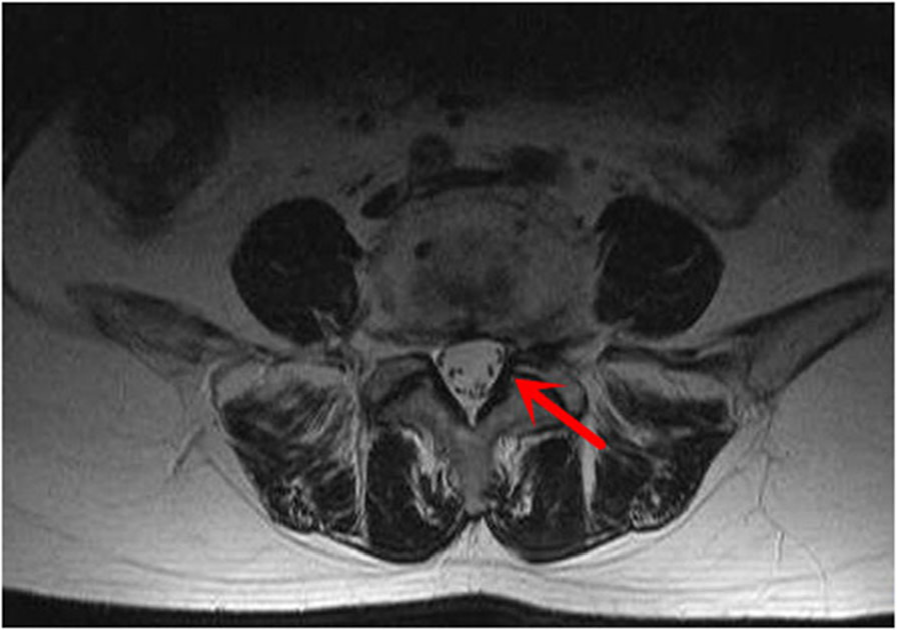
After postoperative 12 months, an MRI scan showed a complete absence of the gas discal cyst at the site of treatment (red arrow)

Of the three patients with gas-filled discal cysts at our hospital, two were females and one was male, with a mean age of 56.7 years (range, 55–60 years). Patient no. 2 had a history of hepatitis. The remaining two patients had no history of any disease. All three patients presented with backache and numbness of the left limb (Table 1). A physical examination found paresthesia of the L5 dermatome of the left foot. The straight leg-raising test was positive in two patients (no. 1 and no. 3). All lesions occurred at the level of L4/5. The preoperative 3-dimensional computed tomography (3D-CT) with discogram and magnetic resonance image (MRI) showed the cyst connected to the corresponding intervertebral disc in all cases (Fig. 1). Gas-filled discal cysts show low-density shadows on 3D-CT. Gas-filled discal cysts are round to oval, extradural masses with low signal intensity on T1-weighted images and T2-weighted images (Fig. 2). Other examinations, including echocardiogram, electrocardiogram, coagulation function, and routine blood examination, were normal. All patients underwent TPED. The mean operative duration was 91.6 min (range, 65–115 min), and the mean blood loss was 26.7 ml (range, 10–50 ml). The mean length of hospital stay was 7.3 days (range, 7–8 days). All patients recovered successfully and were eventually discharged. The median postoperative follow-up duration was 26 months (range, 12–36 months). An MRI scan 3 years postoperative showed a complete absence of a gas-filled discal cyst at the site of treatment in one patient (Fig. 3). Two patients were lost to follow-up.

**Table 1:** Patient characteristics and treatment history in our study

Features	Patient no. 1	Patient no. 2	Patient no. 3
Gender	Male	Female	Female
Age	55	55	60
Presentation	Backache and left lower limb numbness	Backache and left lower limb numbness	Backache and left lower limb numbness
Previous history	No	Hepatitis	No
Level	L4/5	L4/5	L4/5
Direction	Left	Left	Left
Surgical strategy	TPED	TPED	TPED
Operating time (min)	65	115	95
Blood loss (ml)	20	50	10
Postoperative complication	No	No	No
Hospital stay (days)	8	7	7
Follow-up (months)	36	30	12
Recurrence	No	No	No
Current status	NED	NED	NED

*NED* no evidence of disease, *TPED* transforaminal percutaneous endoscopic discectomy

### Published case report findings

We searched the literature from January 1990 to January 2019. According to the titles and abstracts, a total of 20 articles were related. One article was not included in the analysis because there were no relevant data [4, 5]. One article, a letter to the editor, was not included in the analysis [6]. In this letter to the editor, more than 200 cases of intraspinal gas were described. However, this letter contained little or no clinical outcomes. Because of the lack of data, this letter was not included. Eighteen articles were identified from the database searches, and a total of 42 patients were included [7–23] (Table 2). There were 28 males and 14 females. The mean age was 56.8 years, ranging from 27 to 85 years. Lower back pain was the major symptom. Twenty-two patients underwent surgery, 4 patients underwent percutaneous needle aspiration, 2 patients underwent drug therapy, 13 patients received nonoperative treatment, and 1 patient was unknown. Cysts recurred in 2 patients who underwent percutaneous needle aspiration, and they later underwent surgical treatment.

**Table 2:** Clinicopathologic features of gassy disc cyst present in the English literature

	Case	Age (years)	Gender	Site	Symptoms	Treatment	Follow-up (months)	Status
Cebeci [7]	1	55	Female	L4–L5	Low back pain	Unknown	Unknown	Unknown
Firth [8]	1	70	Male	L4–L5	Low back and gluteal pain	Surgical excision	Unknown	Unknown
Jeon [9]	1	76	Male	L4–L5	Bilateral lower leg pain	Surgery	24	NED
Kakitsubata [10]	4	57 27 73 51	Male Female Female Male	L5–S1 L4–L5 L5–S1 L4–L5	Pain in the right lower extremity Right lumbar radiculopathy Pain in the left lower extremity Pain radiating into the right leg	L5–S1 discectomy Analgesics and oral steroids Surgery Percutaneous needle aspiration	12 14 Unknown Unknown	NED NED Unknown Unknown
Kang [11]	1	68	Female	L5–S1	Lower back and radiating pain	Percutaneous needle aspiration	12	NED
Ambesi Impiombato [12]	1	85	Female	L5–S1	Left lumbar sciatica	CT-guided needle aspiration	6	NED
Cho [13]	1	80	Male	L2–L3	Pain in both legs	CT-guided aspiration surgery	1–14	Recurred-NED
Yun [14]	2	83 72	Male Female	L4–L5 L5–S1	Back and left radiating pain Pain in the left lower extremity	Partial hemilaminectomy Left L5–S1 discectomy	6 12	NED NED
Chiu [15]	1	71	Female	L3/L4	Low back pain	Surgical treatment	Unknown	Unknown
Kudo [16]	2	51 66	Female Male	L3/L4 L4–L5	Progressive low back pain Progressive low back pain	Surgery Surgery	12 Unknown	NED Unknown
Qasho [17]	1	55	Male	L4–L5	Pain in the left lower limb	Surgery	1	NED
Kawaguchi [18]	1	60	Male	L3/L4	Low back pain	Surgery	12	NED
Harvey [19]	1	61	Male	L3/L4	Unremitting right-sided sciatica	Cyst excision	Unknown	Unknown
Firth [8]	1	70	Male	L5–S1	Left buttocks pain	Surgical excision	Unknown	Unknown
Fandino [20]	2	48 59	Male Male	L5–S1 L5–S1	Back pain Persistent sciatica	Surgery Surgery	Unknown Unknown	Unknown Unknown
Lin [21]	1	40	Male	L3/L4	Lower back pain	Surgery	Unknown	Unknown
Hidalgo-Ovejero [22]	19	55 56 58 36 36 52 46 44 41 47 58 43 60 47 29 68 45 66 45	Male Female Male Male Female Male Male Male Male Male Female Male Male Male Male Male Female Male Female	L5–S1 L5–S1 L5–S1 L5–S1 L5–S1 L5–S1 L5–S1 L4–L5 L5–S1 L4–L5 L5–S1 L5–S1 L4–L5 L5–S1 L5–S1 L4–L5 L4–L5 L4–L5 L5–S1	Unknown Unknown Unknown Unknown Unknown Unknown Unknown Unknown Unknown Unknown Unknown Unknown Unknown Unknown Unknown Unknown Unknown Unknown Unknown	Nonoperative Nonoperative Surgery Surgery Surgery Nonoperative Nonoperative Nonoperative Nonoperative Surgery Nonoperative Nonoperative Nonoperative Surgery Surgery Nonoperative Nonoperative Nonoperative Nonoperative	Unknown Unknown Unknown Unknown Unknown Unknown Unknown Unknown Unknown Unknown Unknown Unknown Unknown Unknown Unknown Unknown Unknown Unknown Unknown	Unknown Unknown Unknown Unknown Unknown Unknown Unknown Unknown Unknown Unknown Unknown Unknown Unknown Unknown Unknown Unknown Unknown Unknown Unknown
Tobback [23]	1	74	Female	L4–L5 L5–S1	Back pain	Anti-inflammatory medication- Surgery	36 Unknown	Recurred Unknown

*NED* no evidence of disease

## Discussion

Discal cysts are extremely rare lesions, described as cysts with a direct connection with the corresponding intervertebral disc [2]. Discal cysts that contain gas are even more rare. The etiology and pathogenesis of discal cysts remain unknown, but several hypotheses have been proposed [24]. The vascular theory hypothesizes that it is an organized epidural hematoma result of hemorrhage of the epidural venous plexus resulting from disc herniation or preceding discal injury, which develops acutely and later acquires a pseudomembrane [2]. Jeong and Bendo [25] hypothesized that the formation of discal cysts was not a vascular phenomenon but resulted from a change in a herniated disc. Some scholars support the theory that a discal cyst is due to focal degeneration or annular injury of an intervertebral disc producing a corresponding herniated disc with subsequent spilling of fluid from the herniated disc tissue that triggers an abacterial inflammatory response, resulting in the formation of a pseudomembrane and development of a discal cyst [26]. Based on our intraoperative findings, we agree that the underlying etiology and pathogenesis results from an annular injury or focal degeneration, leading to a herniated disc with a subsequent series of reactions resulting in the formation of a reactive pseudomembrane that finally becomes a discal cyst.

Chief complaints, symptoms and signs of discal cysts can be similar to those of patients with typical lumbar disc herniation [1]. The early stage of discal cyst formation is asymptomatic, and no treatment is necessary because the discal cyst puts only small pressure on the canalis spinalis. However, as the discal cyst grows, patients present with different symptoms, including backache and numbness. Other diseases, which can present with similar clinical symptoms including lower back pain and radiculopathy are perineural or Tarlov cysts, epidural hematomas, ligamentum flavum cysts, arachnoid cysts, and synovial cysts [27].

Imaging examinations, including 3D-CT and MRI, are used for assessing discal cysts. Gassy discal cysts show low-density shadows on 3D-CT. The typical findings for fluid discal cysts are round to oval extradural masses with low signal intensity on T1-weighted images and high signal intensity on T2-weighted images [28]. However, this signal depends on the contents of the fluid. When the discal cysts contain gas and no fluid, masses have low signal intensity on T1-weighted images and T2-weighted images.

According to cases in the literature, partial hemilaminectomy, microscopic excision, or endoscopic excision are generally accepted as the definitive and effective treatment of choice for discal cysts. To our knowledge, the largest single-center experience describing the surgical treatment of discal cysts was described by Wang et al. [29]. This author reported the microscopic surgical outcomes of nine patients with symptomatic radiculopathy caused by discal cysts and believed that the operative indications for discal cysts are similar to those of lumbar disc herniation [29]. Although the majority of cases of discal cysts have been treated with surgical resection, computed tomography-guided aspiration has also been described [30, 31]. Endo [27] described a lumbar discal cyst that was treated with computed tomography-guided aspiration and steroid injection. This author believed that corticosteroid injection into the cyst was important for minimizing the risk of recurrence [27]. However, Kang et al. [32] performed similar aspirations without steroid injections, and no patients reported any recurrence of the cysts. Meanwhile, Cho et al. [13] described a gas-filled intradural cyst that was treated with computed tomography-guided aspiration. Unfortunately, the patient’s symptoms recurred 1 month later, and the CT showed re-accumulation of gas in the intradural cyst. The patient underwent open intradural surgery via the posterior approach. Therefore, steroid injection for discal cysts is still controversial. In addition, Demaerel et al. [33] and Takeshima et al. [34] report cases of spontaneous regression of a discal cyst without intervention. In our study, discal cysts were treated effectively by TPED. However, in order to provide more definitive evidence of standard and effective treatment for discal cysts, more studies on diagnostic and therapeutic strategies for discal cysts are needed, and careful analysis and long-term follow-up are necessary.

## Conclusion

Gassy discal cysts are an extremely rare disease and may manifest with symptoms and signs very similar to lumbar disc herniation. TPED is the standard, feasible, effective, and successful treatment of gassy discal cysts, and it should be conducted by an experienced surgeon with awareness of the potential risk of nerve root injury.

## Data Availability

We declare that the materials described in the manuscript, including all relevant raw data, will be freely available to any scientist wishing to use them for noncommercial purposes, without breaching participant confidentiality.

## Abbreviations

TPED: Transforaminal percutaneous endoscopic discectomy
3D-CT: 3-Dimensional computed tomography
MRI: Magnetic resonance imaging
